# Neoadjuvant docetaxel and capecitabine (TX) versus docetaxel and epirubicin (TE) for locally advanced or early her2-negative breast cancer: an open-label, randomized, multi-center, phase II Trial

**DOI:** 10.1186/s12885-022-10439-0

**Published:** 2022-12-28

**Authors:** Houpu Yang, Ling Xu, Shan Guan, Xiaopeng Hao, Zhicheng Ge, Fuzhong Tong, Yingming Cao, Peng Liu, Bo Zhou, Lin Cheng, Miao Liu, Hongjun Liu, Fei Xie, Siyuan Wang, Yuan Peng, Chaobin Wang, Shu Wang

**Affiliations:** 1 Peking University People’s Hospital Breast Center, Beijing, China; 2 Breast Disease Center, Peking University First Hospital, Beijing, China; 3 Department of Breast Center, Beijing Tongren Hospital, Capital Medical University, Beijing, China; 4 Department of General Surgery, First Medical Center of Chinese PLA General Hospital, Beijing, China; 5 Department of General Surgery, Beijing Friendship Hospital, Capital Medical University, Beijing, China

**Keywords:** Breast neoplasms, Neoadjuvant therapy, Anthracycline, Taxane, Capecitabine

## Abstract

**Purpose:**

The combination of taxanes and anthracyclines is still the mainstay of chemotherapy for early breast cancer. Capecitabine is an active drug with a favorable toxicity profile, showing strong anti-tumor activity against metastatic breast cancer. This trial assessed the efficacy and safety of the TX regimen (docetaxel and capecitabine) and compared it with the TE (docetaxel and epirubicin) regimen in locally advanced or high risk early HER2-negative breast cancer.

**Patients and methods:**

This randomized clinical trial was conducted at five academic centers in China. Eligible female patients were randomly assigned (1:1) to the TX (docetaxel 75 mg/m^2^ d1 plus capecitabine 1000 mg/m^2^ twice d1–14, q3w) or TE (docetaxel 75 mg/m^2^ d1 plus epirubicin 75 mg/m^2^ d1, q3w) groups for four cycles. The primary endpoint was a pathological complete response in the breast (pCR). Secondary endpoints included pCR in the breast and axilla, invasive disease-free survival (iDFS), overall survival (OS), and safety.

**Results:**

Between September 1, 2012, and December 31, 2018, 113 HER2-negative patients were randomly assigned to the study groups (TX: *n* = 54; TE: *n* = 59). In the primary endpoint analysis, 14 patients in the TX group achieved a pCR, and nine patients in the TE group achieved a pCR (25.9% vs. 15.3%), with a not significant difference of 10.6% (95% CI -6.0–27.3%; *P* = 0.241). In a subgroup with high Ki-67 score, TX increased the pCR rate by 24.2% (95% CI 2.2–46.1%; *P* = 0.029). At the end of the 69-month median follow-up period, both groups had equivalent iDFS and OS rates. TX was associated with a higher incidence of hand-foot syndrome and less alopecia, with a manageable toxicity profile.

**Conclusion:**

The anthracycline-free TX regimen yielded comparable pCR and long-term survival rates to the TE regimen. Thus, this anthracycline-free regimen could be considered in selected patients.

**Trial Registration:**

ACTRN12613000206729 on 21/02/2013, retrospectively registered.

**Supplementary Information:**

The online version contains supplementary material available at 10.1186/s12885-022-10439-0.

## Background

The introduction of taxanes and anthracyclines as modern chemotherapy has drastically improved the outcomes of patients with early breast cancer (EBC) [1, 2]. The combination of taxanes and anthracyclines has been recommended as the preferred regimen for patients with human epidermal growth factor receptor 2 (HER2)-negative breast cancer, especially those with locally advanced breast cancer (LABC) or high risk EBC, who were pursuing rapid tumor shrinkage through neoadjuvant chemotherapy [3].

However, anthracycline-containing chemotherapy causes quality of life-threatening, long-term side effects, such as heart failure and treatment-related leukemia [2]. As the number of long-term survivors, elderly patients, and patients with cardiac risk factors increases, the toxic profile becomes a more important discriminator in the neoadjuvant/adjuvant treatment decision-making framework, and the de-escalation of anthracycline-containing regimens is gaining popularity in neoadjuvant/adjuvant trials. The US Oncology 9735 trial demonstrated the superiority of four cycles of TC (docetaxel plus cyclophosphamide) to four cycles of AC (doxorubicin plus cyclophosphamide) [4], and the West German Study Group PlanB trial revealed the non-inferiority of six cycles of TC to a standard AC regimen followed by a taxane in clinically high-risk or genomically intermediate- to high-risk HER2-negative patients [5]. The success of the TC regimen in the adjuvant setting increased the confidence to explore efficacy- and toxicity-balanced regimens in EBC.

Capecitabine is a nucleoside analog commonly used in patients with metastatic breast cancer [6, 7]. It is a prodrug of 5-fluorouracil (5-FU) activated by a complicated three-step conversion through three key enzyme activities. The final step of capecitabine conversion to 5-FU requires thymidine phosphorylase, which is highly expressed in breast cancer tissue [8]. Preclinical and clinical studies have shown that docetaxel upregulates thymidine phosphorylase in breast tumor tissue, indicating potential synergy between docetaxel and capecitabine (TX) [8, 9]. Several studies have demonstrated the highly effective anti-tumor activity of the TX regimen in metastatic breast cancer, even in anthracyclines and taxanes pretreated tumors [10, 11]. However, little is known about the feasibility of translating this combination to the neoadjuvant/adjuvant setting.

Here, we performed a randomized phase II trial for locally advanced and high risk HER2-negative breast cancer to assess the efficacy and toxicity of the TX regimen using a combination of docetaxel and epirubicin (TE) as the standard comparator.

## Patients and methods

### Study design and patients

This study was a randomized, open-label, phase II, multi-center clinical trial comparing TX with TE as neoadjuvant chemotherapy for stage II/III breast cancer conducted in Beijing, China. The study was approved by the Peking University Peoples’ Hospital Ethics Committee and Ethics Committees of all participating institutions and was conducted in accordance with the Declaration of Helsinki. Informed consent was obtained from each study subject prior to participating. This study was registered at the Australian New Zealand Clinical Trials Registry (ACTRN12613000206729).

Eligible patients were women aged at least 18 years with clinical-stage cT1c–4/cN0-3/M0 (stage II-III) breast cancer. The histological diagnosis of the primary lesion was obtained at a local laboratory. Hormone receptor (estrogen receptor [ER] and progesterone receptor [PR] status), HER2, and Ki-67 status of the primary tumor had to be known. Participants were required to have an Eastern Cooperative Oncology Group (ECOG) performance status of 0 or 1. Participants were also required to have adequate organ function based on laboratory assessment of absolute neutrophil count, platelet count, hemoglobin, serum creatinine, aspartate transaminase (AST), alanine transaminase (ALT), total serum bilirubin, and serum alkaline phosphatase. Exclusion criteria included stage IV (metastatic) breast cancer, inflammatory breast cancer without in-breast tumor, a history of invasive breast cancer, and previous systemic therapy for the treatment or prevention of breast cancer. Patients were also excluded if they had HER2 + disease and neoadjuvant anti-HER2 targeted therapy would be prescribed.

Eligible patients were randomly assigned to receive either TX or TE (1:1). Patients were randomized with a permuted block randomization scheme using a telephone randomization system with no stratification factor. The generation of the random allocation sequence was completed by the online system. The physicians who were responsible for the participants enrolled the patients and assigned them to interventions.

### Procedures

The TX group received intravenous docetaxel (75 mg/m^2^) on day 1 plus oral capecitabine (1000 mg/m^2)^ twice per day from day 1 to day 14 for every three-week cycle. The TE group received intravenous docetaxel (75 mg/m^2^) and epirubicin (75 mg/m^2^) on day 1 of every three-week cycle. Treatment was administered for four cycles before definitive surgery. Treatment was discontinued if progressive disease or unacceptable toxicity was noted. Pretreatment for docetaxel was performed in accordance with local regulations. Generally, 7.5 mg of oral dexamethasone was administered twice daily for a total of six doses, starting one day before each infusion. Dose reductions were permitted, and prophylactic granulocyte colony-stimulating factor (G-CSF) administration was allowed. Recombinant human G-CSF, filgrastim or biosimilar agents, could be prescribed at a dosage of 4–5 ug/Kg either for primary or secondary prophylaxis of events related to neutropenia. The selection of prophylactic use of G-CSF or not, primary or secondary, and the treatment duration, were decided by the chief physicians.

Definitive surgery was performed within two to four weeks after the last neoadjuvant chemotherapy treatment. The type of breast surgery (mastectomy or breast-conserving surgery) and axillary treatment (sentinel lymph node biopsy or axillary lymph node dissection) were determined by the treating surgeons. After surgery, adjuvant chemotherapy was given at the discretion of the physicians. Radiotherapy and endocrine therapy were recommended according to international guidelines.

### Statistical analysis

The pCR, defined as the absence of residual invasive tumor cells in the breast (ypT0/is), was measured as the primary endpoint. Secondary endpoints included pCR in the breast and axilla (ypT0/isN0), clinical response assessed using Response Evaluation Criteria in Solid Tumors (RECIST ver1.1), clinical and pathologic stage (CPS) and estrogen receptor status and histologic grade (EG) (CPS&EG) score, invasive disease-free survival (iDFS), and overall survival (OS). Treatment-related toxicities were also examined using the National Cancer Institute Common Terminology Criteria for Adverse Events (version 4.0) system.

The sample size was estimated by assuming that the pCR rate of the TX group would be non-inferior to that of the TE group by inferiority margin of 3%. The type I error was set at 5% using two-sided significance tests. With a sample size of 440 patients, there was an 80% probability of rejecting the null hypothesis. The trial was terminated in December 2018 for slow enrollment after 166 patients had been screened. Because the confirmatory analysis was deemed underpowered, we carried out an exploratory analysis to determine the feasibility and safety of the TX regimen in the neoadjuvant setting.

Patients who received the assigned chemotherapy were included in the efficacy analysis. Those who received at least one dose of either regimen were included in the safety analysis. The pCR rates were compared between the two groups using the χ2 or Fisher’s exact test. Differences and 95% confidence intervals (CIs) were calculated. The pCR rates of the groups were compared for different Ki-67 levels and various molecular subtypes. The subtype definition was in accordance with current guidelines, while the Ki-67 score was categorized into high and low with a threshold of 20%. Clinical response was assessed using RECIST criteria v1.1. The CPS&EG score was developed to evaluate the response to neoadjuvant therapy after incorporating clinical stage, pathological stage, ER, and grade and validated to predict long-term survival. The CPS&EG scores were compared between the groups using the χ2 test. iDFS and OS were estimated using the Kaplan–Meier method and compared between groups using the log-rank test. Safety profiles were compared using the χ2 or Fisher's exact test.

The final date of data acquisition for this study was December 28, 2021. Data were analyzed with R (http://r-project.org).

## Results

### Patient and tumor characteristics

**Fig. 1:**
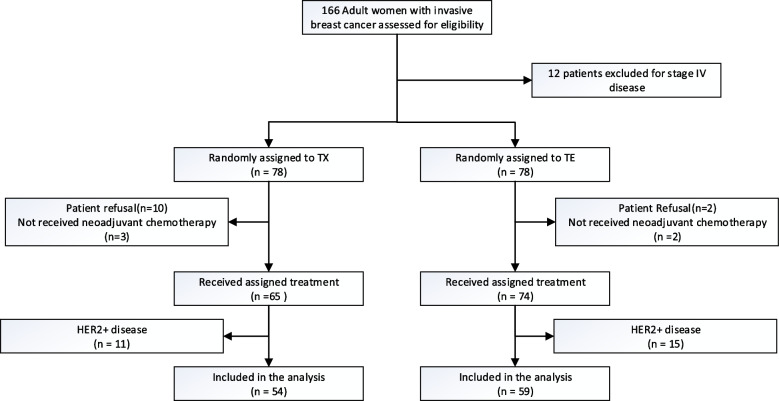
CONSORT diagram of patient disposition. TX, docetaxel and capecitabine; TE, docetaxel and epirubicin

Between September 1, 2012, and December 31, 2018, 139 patients from five participating centers in China were randomly assigned to the treatment groups (TX: *n* = 65; TE: *n* = 74). Because anti-HER2 targeted therapy was not widely used in China for medical resource reasons at the time of the trial design, we included patients who would not have received trastuzumab in the neoadjuvant phase at the start of the study. With improvements in the Chinese public medical insurance reimbursement policy for anti-HER2 therapy, we stopped recruiting HER2 + patients in 2016. Finally, we excluded the 26 HER2 + patients from the final analysis dataset for confounding concerns. Thus, 113 patients were evaluated for the primary endpoint (TX: *n* = 54; TE *n* = 59). Figure 1 shows the CONSORT study flowchart. The demographic characteristics of the 113 subjects who received the assigned treatments are presented in Table 1. Patient and treatment features were balanced between the two groups. The median age of the overall population was 52 (22–79 years). TX group included more old patients (> 50: 61.1% vs 42.2%) than TE group, with a marginal significance (*P* = 0.072). T2-4 tumors were found in 78.8% of the study patients, and 89.4% of the axillary lymph nodes were positive. The proportions of breast-conserving surgery in the two groups were 20.4% and 18.6%, respectively (*P* = 1.000). In the TE group, 34 patients (45.9%) received primary prophylaxis with G-CSF from their first cycles and 23 patients (31.1%) received secondary prophylaxis after an episode of febrile neutropenia or grade 4 neutropenia. In the TX group, no patient received primary prophylaxis and 19 (29.2%) patients received secondary prophylaxis. Dose was reduced in 8 patients (14.8%) in TX group and 9 patients (15.3%) in TE group respectively. More dose reduction happened in patients older than 50 (11 patients in age > 50 group vs 6 patients in ≤ 50 group). A total of 84.1% of study patients (TX: 92.6%; TE: 83.1%) received response-guided adjuvant therapy at the discretion of the treating physicians. The most common adjuvant chemotherapy for TX group was additional two to four cycles of TX and an additional AC regimen. Most patients in TE group were given additional two to four cycles of TE to complete a full course of TE.

**Table 1:** Patient and Tumor Characteristics

	Total (*n* = 113) N (%)	TX (*n* = 54) N (%)	TE (*n* = 59) N (%)	p
Age				
> 50	58 (51.3)	33 (61.1)	25 (42.4)	0.072
≤ 50	55 (48.7)	21 (38.9)	34 (57.6)	
Histological Type				
No special type	99 (87.6)	47 (87.0)	52 (88.1)	0.853
Invasive lobular	7 (6.2)	4 (7.4)	3 (5.1)	
Mixed	7 (6.2)	3 (5.6)	4 (6.8)	
Initial cT Stage				
cT0	1 (0.9)	0 (0.0)	1 (1.7)	0.704
cT1	23 (20.4)	9 (16.6)	14 (23.7)	
cT2	70 (61.9)	36 (66.7)	34 (57.6)	
cT3	9 (8.0)	5 (9.3)	4 (6.8)	
cT4	10 (8.8)	4 (7.4)	6(10.0)	
Initial cN Stage				
N0	12 (10.6)	7 (13.0)	5 (8.5)	0.730
N1	46 (40.7)	23 (42.6)	23 (39.0)	
N2	44 (38.9)	20 (37.0)	24 (40.7)	
N3	11 (9.7)	4 (7.4)	7 (11.9)	
Hormone Receptor				
Negative	35 (31.0)	15 (27.8)	20 (33.9)	0.618
Positive	78 (69.0)	39 (72.2)	39 (66.1)	
Ki-67				
> 20%	72 (63.7)	33 (61.1)	39 (66.1)	0.722
≤ 20%	41 (36.3)	21 (38.9)	20 (33.9)	
Breast Surgery				
Breast conservation	22 (19.5)	11 (20.4)	11 (18.6)	1.000
Mastectomy	91 (80.5)	43 (79.6)	48 (81.4)	
Adjuvant Chemotherapy	95 (84.1)	48 (92.6)	47 (83.1)	0.181
Original neoadjuvant regimen	64 (64.5)	23 (47.9)	41 (87.2)	
Non-cross resistant regimen				
Anthracycline + CTX	17 (17.1)	17 (35.4)	0 (0)	
Capecitabine alone	6 (6.1)	1 (2.1)	5 (10.6)	
Others	8 (8.1)	7 (14.6)	1 (2.1)	
Adjuvant Radiotherapy				
Yes	109 (96.4)	52 (96.3)	54 (91.5)	0.441
No	4 (3.6)	2 (3.7)	5 (8.5)	
Adjuvant Endocrine Therapy	76 (67.3)	37 (68.5)	39 (66.1)	0.784
TAM	19 (16.8)	9 (16.7)	10 (17.0)	
AI	48 (42.5)	25 (46.3)	23 (39.0)	
OFS + TAM / AI	9 (8.0)	3 (5.6)	6 (10.1)	

Abbreviations: *CTX* Cyclophosphamide, *TAM* Tamoxifen, *AI* Aromatase Inhibitor, *OFS* Ovarian Function Suppression

### Pathological and clinical response

**Fig. 2:**
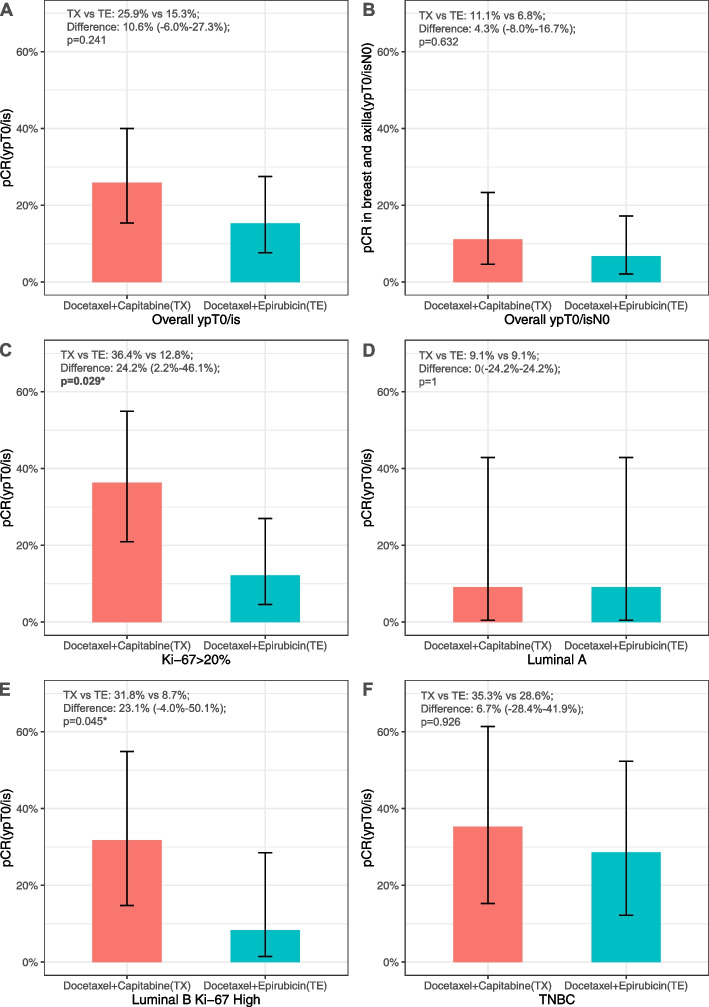
Pathological response **A.** pCR in the breast of overall patients, **B**. pCR in the breast and axilla of overall patients, **C**. pCR of patients with Ki-67 ≥ 20%; **D**. pCR of patients with luminal A disease, **E**. pCR of patients with luminal B Ki-67 high disease; F. pCR of patients with TNBC disease. pCR, pathological complete response; TE, docetaxel and epirubicin; TX, docetaxel and capecitabine

In the primary endpoint analysis, a pCR was achieved by 14 patients in the TX group (25.9%, 95% CI 16.1%–38.9%) and nine patients in the TE group (15.3%, 95% CI 8.2%–26.5%); the difference between the groups was not significant (10.7%, 95% CI -4.2%–25.5%, *P* = 0.241) (Fig. 2A). There was also no significant difference between the groups for the pCR rate in both breast and axilla (Fig. 2B).

**Fig. 3:**
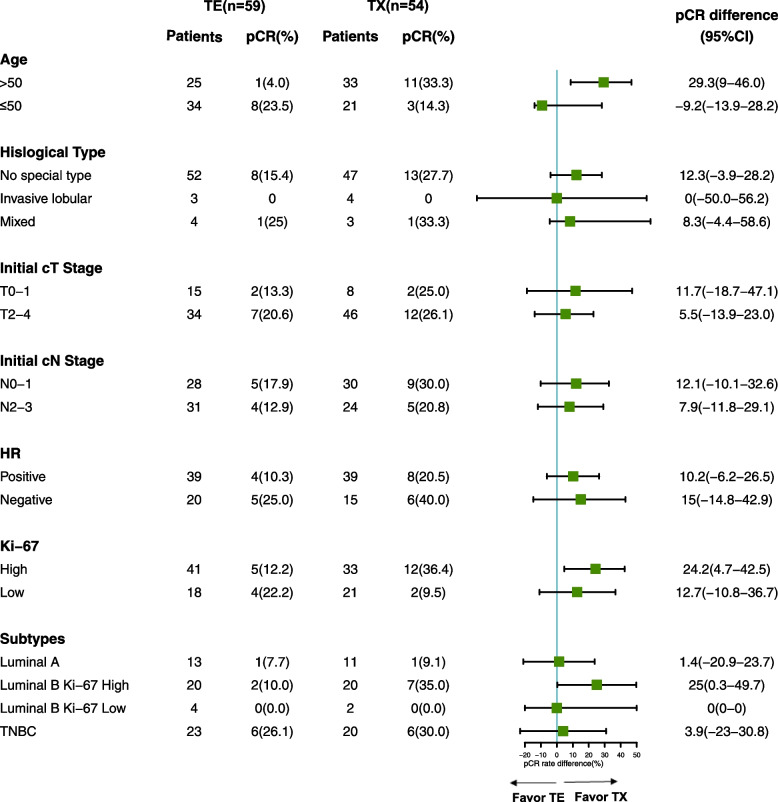
Exploratory subgroup analysis of the two regimens for pathological complete response (pCR) TE, docetaxel and epirubicin; TX, docetaxel and capecitabine; HR, hormone receptor

An exploratory molecular subgroup analysis was performed to find the potential benefit sub-population of TX. The pCR rates were 36.4% (12/33) and 12.8% (5/39) in the Ki-67 high subgroups of the TX and TE groups, respectively (95% CI 3.7%–42%; *P* = 0.026). We further analyzed the other molecular subtype subgroups and found very low pCR rates in the luminal A and luminal B PR negative subtypes. In the luminal B Ki-67 high and triple-negative breast cancer (TNBC) subtypes, TX provided a higher pCR rate; however, statistical significance was only achieved with the luminal B Ki-67 high group. These data are presented in Fig. 2C–F. Additional exploratory subgroup analyses are shown in Fig. 3 as a forest plot. For age subgroups, pCR rate favored TX in age > 50 subgroup, while pCR difference was not significant in patients ≤ 50 years old. The pCR rate difference in different histological types, initial cT stage, initial cN stage, or HR status subgroups were not significant. For molecular subtypes, pCR rate favored TX for Ki-67 high group and luminal B Ki-67 high group as abovementioned.

We also analyzed the clinical response using RECIST and CPS&EG. There was no progressive disease in either group. There were no statistically significant differences in the clinical responses or CPS&EG scores between the two treatment groups (Supplemental Table 1).

### Survival analysis

**Fig. 4:**
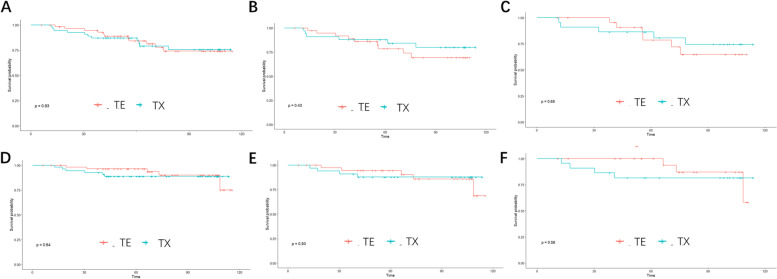
iDFS and overall survival. **A** iDFS for the overall population, **B** iDFS for patients with high Ki-67, **C** iDFS for Luminal B Ki-67 high patients, **D** OS for the overall population, **E** OS for patients with high Ki-67; F. OS for Luminal B Ki-67 high patients

At the end of the 69-month median follow-up period, iDFS and OS were similar between the two groups. The five-year iDFS rates were 87% (95% CI 78.5–96.5%) for TX and 84.2% (95% CI 74.6–95%) for TE (*P* = 0.93) (Fig. 4A). The five-year OS rates were 88.8% (95% CI 80.7–97.7%) for treated with TX compared with 96.4% (95% CI 91.5–100%) when treated with TE (*P* = 0.64) (Fig. 4D). The survival differences between the different regimens were not significant for the Ki-67 high and luminal B Ki-67 high subgroups (Fig. 4B-C, E–F).

### Toxic effects

Both regimens caused a relatively high incidence of neutropenia (TX: 64.6%; TE: 78.4%). The capecitabine-containing regimen increased the incidence of hand-foot syndrome (any grade: 64.6%; grade 3: 20%). The incidence of alopecia was lower in the TX group (18.5% vs. 70.3% for grades 3 and 4). No symptomatic cardiac events were documented for either group during the follow-up period. There was one occurrence of acute lymphocyte leukemia in the TE group. The incidences of other adverse events were comparable between the two groups (Table 2).

**Table 2:** Adverse Effects

	TX (*n* = 65)		TE (*n* = 74)	
	Any Grade (%)	Grade 3–4 (%)	Any Grade (%)	Grade 3–4 (%)
Neutropenia	42(64.6)	18(47.6)	58(78.4)	25(33.8)
Anemia	15(23.1)	1(1.5)	18(24.3)	3(4.1)
Hand-foot syndrome	42(64.6)	13(20)	6(8.1)	0
Sensory neuropathy	27(41.6)	2(3.1)	34(45.9)	1(1.4)
Symptomatic heart failure	0	0	0	0
ALT/AST increased	12(18.5)	5(7.7)	20(27)	9(12.2)
Vomiting	18(27.7)	4(6.2)	22(29.7)	5(6.8)
Nausea	33(50.7)	3(4.6)	38(51.4)	5(6.8)
Diarrhea	12(18.5)	5(7.7)	10(13.5)	3(4.1)
Alopecia	50(76.9)	NA	68(91.9)	NA
Wound infection	3(4.6)	1(1.5)	2(2.7)	0
Leukemia	0	0	1	1(1.4))

## Discussion

The present study was a multi-center prospective randomized controlled study to assess the efficacy and toxicity of four cycles of TX compared to the traditional TE regimen. For HER2-negative patients, the pCR rates of the two regimens were comparable (pCR in the breast: 25.9% vs. 15.3%, *P* = 0.241), with similar results for pCR in both breast and axilla. In a subgroup of patients with a high Ki-67 index, the pCR rate of TX was significantly higher than that of TE (pCR in the breast: 38.4% vs. 12.8%, *P* = 0.029). At the median of the 69-month follow-up, the iDFS and OS were similar between the two groups. Our study showed that TX was a highly active anthracycline-free regimen for breast cancer without compromising long-term survival. It revealed that TX could be a potential choice for patients eligible for neoadjuvant chemotherapy but ineligible for the anthracycline-containing regimen.

Capecitabine has been proved to be effective and well tolerated in metastatic breast cancer [10, 11], and at least 14 trials have explored the introduction of capecitabine into the adjuvant phase. GeparQuattro [12], US Oncology 01,062 [13], FinXX [14], et al., used the additional capecitabine to the standard regimen, while other trials like GEICAM/2003–10 [15] and CALGB 49,907 [16] took capecitabine as substitution, CREATE-X used a neoadjuvant platform to select high-risk non-PCR patients to escalate treatment [17], and in more contemporary studies, CBCSG10 and SYUCC001 restricted its usage in the TNBC subtype [18, 19]. Several meta-analyses generated similar results [20, 21], which showed the addition of capecitabine did not improve DFS or OS in unselected patients, but survival was significantly increased in the TNBC subgroup.

Little is known about the feasibility and efficacy of capecitabine in neoadjuvant setting, and a Korean group proposed substituting anthracycline with capecitabine (TX) to balance the benefit and adverse effects [22]. In 209 stage II–III patients, TX increased the pCR in the primary tumors (21% vs. 10%, respectively, *P* = 0.024) compared with AC. However, the AC control was considered a pretty weak regimen, therefore resulted in some uncertainty about the use of TX in high-risk patients. Our study adopted concurrent taxane and anthracycline as a control and found that the pCR rate, clinical response, and long-term survival were comparable between the two groups. These results indicated that TX could be a potential active choice in the neoadjuvant setting with balanced efficacy and toxicity.

In the subgroup analysis, we found that TX achieved higher pCR in Ki-67 high patients, especially in luminal B Ki-67 high patients, for whom conventional neoadjuvant chemotherapy yielded a pCR rate around 6%–11% [23] and similarly low pCR rate was detected when treated with endocrine therapy combined with CDK4/6 inhibitors [24]. In our study, the pCR rate was 38.4% in patients with luminal B Ki-67 > 20% receiving the neoadjuvant TX regimen, suggesting that TX could be a promising treatment for this subgroup. Ki-67 is a proliferation index, and previous studies have shown that higher Ki-67 levels were associated with poorer prognosis and predictive of better chemotherapy response [20–28]. The mechanism of high capecitabine sensitivity for tumors with a high proliferation rate is still unclear. However, the Ki-67 index is associated with thymidine phosphorylase expression [29], which is a key activation enzyme for capecitabine [30]. Furthermore, capecitabine has a lower impact on the bone marrow-derived immune system and might be an immune modulator [31]. This feature could explain its lower hematological toxicity and the feasibility of two-week continuous administration. We hypothesized that these characteristics allow capecitabine to continuously suppress tumor cells and act as an immune modulator in the tumor microenvironment essential for highly proliferating tumors. However, more data is needed to support these findings and our hypothesis.

Patients with TNBC have a poor prognosis due to its aggressive nature and the lack of endocrine and traditional anti-HER2-targeted therapy [32]. Recently, platinum [33], PD-1/PD-L1 inhibitor [34] and poly (ADP-ribose) polymerase inhibitors (PARPi) [35] have been integrated into standard chemotherapy. However, these new escalation treatments are associated with specific short-term and long-term adverse effects, suggesting tolerance could be a problem for future decision-making. Our study showed that the pCR rate of neoadjuvant TX for TNBC was 35.3%, a meaningful response for this subtype. Thus, TX might be a neoadjuvant option for selected TNBC patients.

It’s interesting to find that older patients (> 50) were more likely to benefit from TX regimen, while in younger patients TE appears to be as effective as TX. In the current study, there was a trend that dose reduction was more frequent in older patients than in younger patients, which might be the possible reason of the age-related efficacy difference. It was well known that the efficacy of anthracyclines was dose dependent and a higher dose of epirubicin (100 mg/m^2^) was more effective than a lower dose of that (50 mg/m^2^) [36]. However, the response and the dose for capecitabine were not so apparently correlated and significant anti-tumor activity was observed when given at very low dose [37]. In addition, the small sample size and the slight age imbalance might also be potential confounding factors responsible for the efficacy difference.

It is not surprising that TX caused a higher incidence of hand-foot syndrome (20%), and less alopecia, with a manageable toxicity profile, which was consistent with a previous trial on metastatic disease [10]. Unexpectedly, grade 3–4 neutropenia was as frequently observed in the TX group as in the TE group. It was speculated that the physicians were reluctant to administer prophylactic G-CSF in the TX group due to the continuous use of capecitabine, which brought us to an important point that more attention should be devoted to the hematologic toxicity management. The advantage of TX is expected to be less likely to cause rare severe long-term adverse effects, such as heart failure and secondary cancer, which may be observed in larger cohorts. We did not detect any symptomatic cardiac events in either group. However, one patient in the TE group was diagnosed with acute leukemia during follow-up.

There were several limitations to our study due to the early termination of the trial, the pretty small sample size, and the underpowered statistical analysis. However, the results for specific subtypes were still attractive and warrant further study.

Despite its limitations, our study was the first randomized, positive-controlled study to validate the feasibility of using TX as an anthracycline-free regimen against LABC and high risk early HER2-negative breast cancer. This regimen could be an active option for selected patients. These findings must be confirmed in a definitive trial with a larger sample size.

## Supplementary Information

**Additional file 1.**

## Data Availability

The datasets generated during and/or analysed during the current study are not publicly available due to ethics requirements but are available from the corresponding author on reasonable request.
