# Supplementary material for: Neoadjuvant docetaxel and capecitabine (TX) versus docetaxel and epirubicin (TE) for locally advanced or early her2-negative breast cancer: an open-label, randomized, multi-center, phase II Trial
Source: BMC Cancer. 2022 Dec 28;22:1357. doi: 10.1186/s12885-022-10439-0 (PMC9795638; doi:10.1186/s12885-022-10439-0)
Supplement: Supplementary file 1 — Additional file 1. [file 12885_2022_10439_MOESM1_ESM.docx]

Supplementary table 1. Clinical Pathological Response

|  | TX (n = 54) | TE (n = 59) | p |
| --- | --- | --- | --- |
|  | N (%) | N (%) |  |
| RECIST |  |  |  |
| CR | 5 ( 9.3) | 11 (18.6) | 0.350 |
| PR | 41 (75.9) | 41 (69.5) |  |
| SD | 8 (14.8) | 7 (11.9) |  |
| PD | 0 | 0 |  |
| CPS & EG |  |  |  |
| 0–3 | 31 (57.4) | 31 (52.5) | 1.000 |
| > 3 | 23 (42.6) | 28 (47.5) |  |
